# Potential contribution of floral thermogenesis to cold adaptation, distribution pattern, and population structure of thermogenic and non/slightly thermogenic *Symplocarpus* species

**DOI:** 10.1002/ece3.10319

**Published:** 2023-07-15

**Authors:** Mitsuhiko P. Sato, Ayumi Matsuo, Koichi Otsuka, Kohei Takenaka Takano, Masayuki Maki, Kunihiro Okano, Yoshihisa Suyama, Yasuko Ito‐Inaba

**Affiliations:** ^1^ Kazusa DNA Research Institute Kisarazu Japan; ^2^ Kawatabi Field Science Center, Graduate School of Agricultural Science Tohoku University Osaki Japan; ^3^ Tomono‐Kai Society of Nagano Environmental Conservation Research Institute Nagano Japan; ^4^ Natural Environment Division Nagano Environmental Conservation Research Institute Nagano Japan; ^5^ Botanical Gardens Tohoku University Sendai Japan; ^6^ Graduate School of Life Sciences Tohoku University Sendai Japan; ^7^ Department of Biological Environment Akita Prefectural University Akita Japan; ^8^ Department of Agricultural and Environmental Sciences, Faculty of Agriculture University of Miyazaki Miyazaki Japan

**Keywords:** cold Adaptation, ecological niche modeling, Phylogeography, *Symplocarpus*, thermogenic plant

## Abstract

The genus *Symplocarpus* in basal Araceae includes both thermogenic and non/slightly thermogenic species that prefer cold environments. If floral thermogenesis of *Symplocarpus* contributes to cold adaptation, it would be expected that thermogenic species have a larger habitat than non/slightly thermogenic species during an ice age, leading to increased genetic diversity in the current population. To address this question, potential distribution in past environment predicted by ecological niche modeling (ENM), genetic diversity, and population structure of chloroplast and genome‐wide single nucleotide polymorphisms were compared between thermogenic *Symplocarpus renifolius* and non/slightly thermogenic *Symplocarpus nipponicus*. ENM revealed that the distribution of *S. nipponicus* decreased, whereas that of *S. renifolius* expanded in the Last Glacial Maximum. Phylogeographic analyses have shown that the population structures of the two species were genetically segmented and that the genetic diversity of *S. renifolius* was higher than that of *S. nipponicus*. The phylogenetic relationship between chloroplast and nuclear DNA is topologically different in the two species, which may be due to the asymmetric gene flow ubiquitously observed in plants. The results of this study imply that floral thermogenesis of *Symplocarpus* contributes to expanding the distribution during an ice age, resulting in increased genetic diversity due to cold adaptation.

## Graphical abstract

Floral thermogenesis is an interesting and important field in evolutionary biology and plant ecology. This is the first study to compare thermogenic and non‐thermogenic plants and explain floral thermogenesis by evolutionary ecology through ecological niche modeling and phylogeographic analysis of chloroplast and genome‐wide single nucleotide polymorphisms.

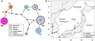

## 1 INTRODUCTION

Thermogenic plants are defined as plants that can increase temperature in their reproductive organs (e.g., inflorescences, flowers, and cones) to at least 0.5°C above ambient temperature (Maekawa et al., 2022). Based on this definition, at least 90 plant species have been reported as thermogenic, half of which are gymnosperm cycads (Ito‐Inaba et al., 2019; Tang, 1987), and the remaining half are angiosperms comprising mostly aroids (Araceae) and a few species of Nymphaeaceae, Magnoliacea, and Nelumbonaceae (Seymour, 2010). Thermogenic aroids are independently observed in the subfamilies Orontioideae (basal Araceae with hermaphroditic flowers) and Aroideae (derived with monoecious flowers). Orontioideae includes only three genera (*Symplocarpus*, *Lysichiton*, and *Orontium*) that grow in northern temperate regions, such as eastern Asia and North America (Mayo et al., 1997). Among these, only *Symplocarpus* displays thermogenic features (Ito‐Inaba et al., 2009). On the contrary, Aroideae is the major taxa of thermogenic aroids and contains many species with high heat‐producing abilities. Floral thermogenesis is widely suggested to promote the dispersal of volatiles that may attract pollinators (Angioy et al., 2004; Gonçalves‐Souza et al., 2017; Meeuse & Raskin, 1988), provide energetic benefits to adult insects (Seymour et al., 2003), facilitate fertilization (Li & Huang, 2009), and prevent freeze damage in plants (Knutson, 1974). However, evolutionary and ecological backgrounds remain unclear, such as how thermogenic plants have evolved adaptively or neutrally, why various plants have acquired thermogenesis in parallel, and if the benefits for plants are worth the energy consumption.

Among the thermogenic aroids, *Symplocarpus* is the only genus that favors inhabiting relatively cool temperate region. Several *Symplocarpus* species, such as American and Asian skunk cabbages (*S. foetidus* and *S. renifolius*, respectively), flowers upon thawing in early spring, maintain the spadix temperature at around 20°C despite below‐freezing ambient air temperature for approximately 1 week (Knutson, 1974; Uemura et al., 1993). A previous study suggests the possibility that thermogenesis at low temperatures contributes to freezing avoidance by maintaining a high respiratory rate (Knutson, 1974). However, it remains largely unknown whether such floral thermogenesis contributes to survival or reproductive success in cold environment, particularly whether it is an adaptive trait. The markedly high heat‐producing ability of *Symplocarpus* species is attributed to the increased cellular respiration facilitated by mitochondrial energy‐dissipating proteins, such as alternative oxidase and uncoupling protein; however, this mechanism of heat generation comes at the cost of reduced ATP production (Ito‐Inaba, Hida, Ichikawa, et al., 2008; Ito‐Inaba, Hida, Mori, et al., 2008; Meeuse, 1975; Wagner et al., 2008). Therefore, given the high cost of floral thermogenesis, it can hardly be expected that the species will evolve entirely neutrally.

Currently, the genus *Symplocarpus* comprises six species (*S. foetidus*, *S. renifolius*, *S. nipponicus*, *S. nabekuraensis*, *S. koreanus*, and *S. egorovii*), half of which have been newly identified and described in the last 20 years (Lee et al., 2021; Otsuka et al., 2002; Pavlova & Nechaev, 2005). As mentioned above, *S. foetidus* and *S. renifolius* are well‐known for their remarkable heat‐producing abilities (Ito‐Inaba et al., 2009; Knutson, 1974). Thermogenesis in the spadix has been previously reported in *S. nabekuraensis* and *S. koreanus* (Lee et al., 2021; Otsuka et al., 2011). Thermogenesis in *S. nipponicus* is rare or inexistent, and the individual size of this species is approximately one‐third of *S. renifolius* (Otsuka et al., 2011). Meanwhile, thermogenesis in *S. egorovii* remains unknown. Among these species, *S. renifolius*, *S. nabekuraensis*, and *S. nipponicus* grow naturally in Japan and its surrounding areas. *S. nipponicus* and *S. renifolius* are widely distributed in these areas, whereas *S. nabekuraensis* is found only in higher altitude (Otsuka, 2002). From a phylogeographical perspective, it is of great interest that closely related species with different heat‐producing abilities co‐inhabit similar regions within Japan and its surrounding areas.

The common ancestor of the members of *Simplocarpus* diverged 8.8 million years ago and was independently exposed to past climate change (Lee et al., 2019). One clade underwent differentiation into thermogenic species (*S. renifolius*, S. *nabekuraensis*, and *S. foetidus*), and another clade underwent differentiation into non/slightly/unknown‐thermogenic species, *S. nipponicus* and *S. egorovii*. If floral thermogenesis contributes to cold adaptation in *Symplocarpus*, ecological and/or evolutionary consequences should be considered. For example, if distributions between thermogenic and non/slightly thermogenic species in East Asia were different during an ice age, thermogenic species would expand to more northern and colder regions and distribute sporadically to suitable habitats or refugia. Consequently, thermogenic species would have higher genetic divergence and clearly diverged population structures in the process where both species are distributed to the current areas.

To address this, we compared ecological niche modeling (ENM) predictions of potential distribution due to past climate change, niche divergence, genetic diversity, and population structure analysis of chloroplast DNA (cpDNA) and genome‐wide single nucleotide polymorphisms (SNPs) between thermogenic *S. renifolius* and non/slightly thermogenic *S. nipponicus*. *S. nabekuraensis* was omitted from the comparison since it is found in very limited areas and has a relatively small population. Our findings imply that floral thermogenesis contributes to the cold adaptation in *Symplocarpus*.

## 2 MATERIALS AND METHODS

### 2.1 Sample collection

Leaves of five *Symplocarpus* species and *Lysichiton camtschatcensis* (outgroup) were collected from Japan in 2009, 2010, 2012, 2019, and 2020 for DNA extraction. *Symplocarpus* leaves were also obtained from museum specimen at Tohoku University Botanical Gardens and Nagano Environmental Conservation Research Institute. A total of 154 samples from 96 individuals of *S. renifolius*, 34 of *S. nipponicus*, 9 of *S. nabekuraensis*, 9 of *S. egorovii*, 2 of *S. foetidus*, and 4 of *L. camtschatcensis* were collected for cpDNA analysis. A total of 227 samples from 135 *S. renifolius* individuals and 92 *S. nipponicus* individuals were collected for nuclear DNA analysis. Leaf samples collected in 2009, 2010, and 2012 were stored at −20°C, and those collected in 2019 and 2020 were dried using silica gel and stored at room temperature.

### 2.2 Data and distribution modeling

**FIGURE 1:**
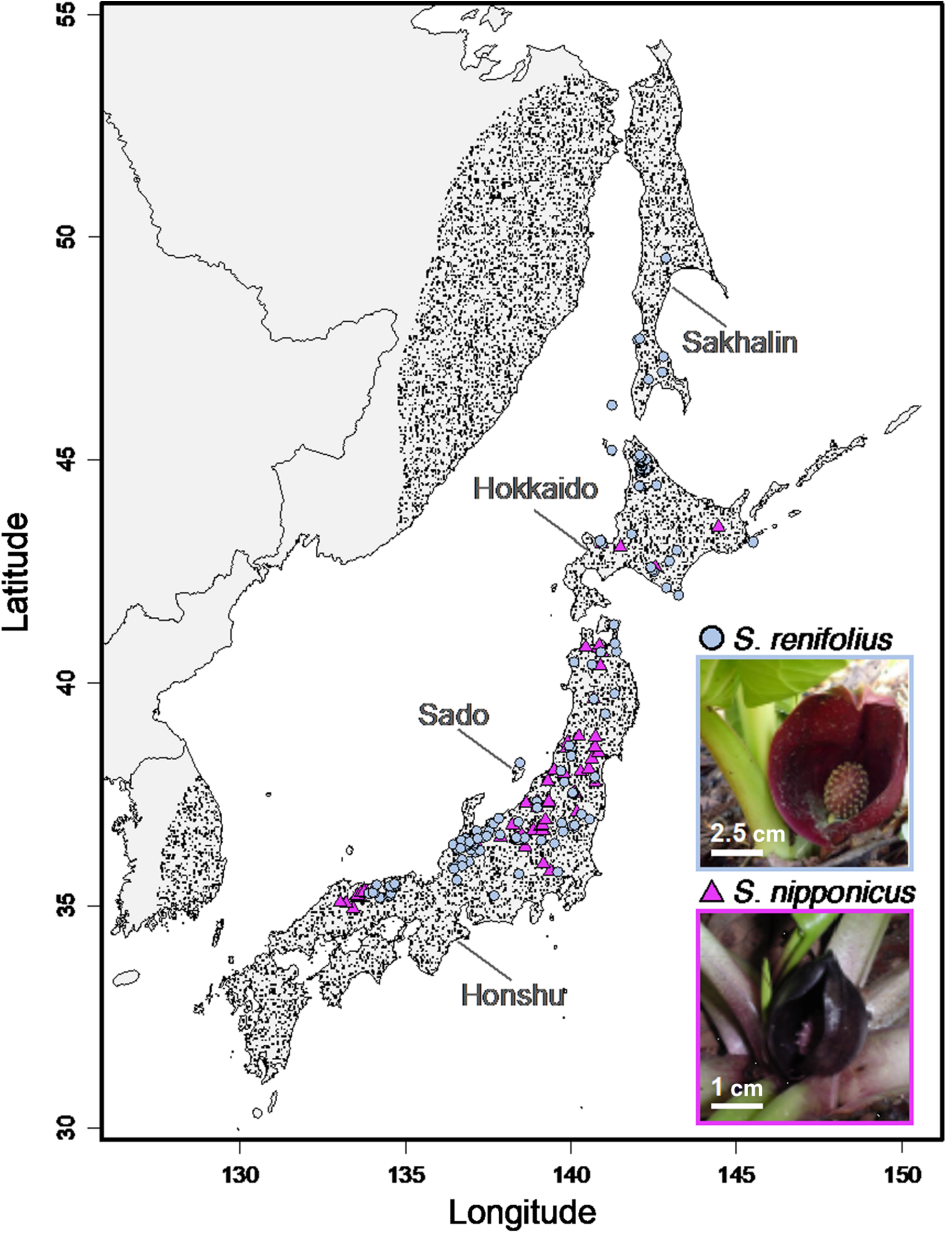
Occurrence sites of *Symplocarpus renifolius* and *Symplocarpus nipponicus* in Japan, Sakhalin, and Northeast Asia obtained from GBIF database, museum specimen, and sampling points for this study. Black dots indicate background sites using ecological niche modeling.

Occurrence data for *S. renifolius* and *S. nipponicus* were obtained from the sampling locations and specimen information from the Tohoku University Botanical Gardens and downloaded from the Global Biodiversity Information Facility (GBIF.org, 12 November 2020) (Figure 1). All records from museum specimens and GBIF were carefully checked against satellite images on Google Maps (https://www.google.com/maps) and erroneous location data were removed. Duplicated location data were also removed. Total location data in Japan and southern Sakhalin of 161 points for *S. renifolius* and 69 points for *S. nipponicus* were obtained.

Current environmental data of 19 bioclimatic variables and past environmental data from the Mid‐Holocene (MH) and Last Glacial Maximum (LGM) were retrieved from WorldClim 1.4 (Hijmans et al., 2005) at a resolution of 2.5 arc minutes. Environmental data for MH and LGM were estimated using nine (BCC‐CSM1‐1: BCMH, CCSM4: CCMH, CNRM‐CM5: CNMH, HadGEM2‐CC: HGMH, HadGEM2‐ES: HEMH, IPSL‐CM5A‐LR: IPMH, MIROC‐ESM: MRMH, MPI‐ESM‐P: MEMH, and MRI‐CGCM3: MGMH) and three models (CCSM4: CCLGM, MIROC‐ESM: MRLGM, MPI‐ESM‐P: MELGM), respectively. Five (bio02: Monthly Mean Diurnal Range, bio08: Mean Temperature of Wettest Quarter, bio10: Mean Temperature of Warmest Quarter, bio13: Precipitation of Wettest Month, bio19: Precipitation of Coldest Quater) of the 19 environmental variables were selected based on variance inflation factors for random points around 500 km of occurrence locations to avoid strong collinearity. This was performed using the vifstep function of the usdm package (version 1.1‐18) in R. Some MH (HEMH, HGMH, IPMH, MRMH, and MGMH), and LGM (CCLGM and MPLGM) data were removed since five environmental variables of these climate models had extrapolation around Japan (Figure S1).

We then aimed to select the best modeling method of five, which included Generalized Linear Model (GLM), Generalized Additive Model (GAM; Guisan et al., 2002), Maximum Entropy Model (Maxent; Phillips & Dudík, 2008), Random Forest (RF; Breiman, 2001), and Support Vector Machine (SVM; Drake et al., 2006), to predict the potential current distribution of *S. renifolius* and *S. nipponicus* (Figure S2). GLM and GAM are modeling methods based on regression analysis, while Maxent, RF, and SVM are based on machine‐learning techniques. All models were fitted with five environmental variables before removing collinearities. In the Maxent parameter, the regularization multiplier beta was fitted from 1 to 10, with one as the best parameter, and the feature class was set by default automatic configuration. RF and SVM were tuned using tuneRF function of the randomForest package version 4.16‐14 and tune.svm function of the e1071 package version 1.7‐9 in R, respectively. The performance of these models was evaluated using the dismo package version 1.3‐8 in R with five predictability indices: area under the receiver operating characteristic curve (AUC) (Swets, 1973), accuracy ([true positives + true negatives]/total *n*), sensitivity (true positives/[true positives + false negatives]), specificity (true negatives/[false positives + true negatives]), and informedness (sensitivity + specificity − 1) (Figure S2). The results showed that the best modeling methods were RF in *S. renifolius* and Maxent in *S. nipponicus* according to AUC, although none of the methods showed remarkably high performance. For interspecific comparison of the possible distribution range and factors determining the distribution, the Maxent method for each species was uniformly selected, but RF was not. Maxent has been previously applied to closely related species, such as *S. foetidus* (Kim et al., 2018), and to species with small sample sizes and widespread distributions (Blair et al., 2013; Fuchs et al., 2018; van Proosdij et al., 2016). Assuming temporal stability of the ecological niche for both species, the constructed model was applied to LGM and MH climatic layers to predict the past distributions of both species.

Niche breadth and similarity between predicted niches were measured by Levin's niche breadth (Levins, 1968), Schoener's *D* (Schoener, 1968), and *I* (Warren et al., 2008) using ENMTools package, version 1.0.5 (Warren et al., 2021), in R. The differences in the niche between *S. nipponicus* and *S. renifolius* were evaluated by niche identity test and symmetric background test using ENMTools. The niche identity test addresses whether a pair of closely related species are effectively identical in their realized environmental distributions using null distribution constructed from actual occurrent points that are randomly reassigned. The symmetric background test addresses whether different environmental distributions reflect any underlying divergence in ecological tolerances or preferences using null distribution constructed from randomly chosen points from the broad region where both species live. For this test, random points around 500 km from the point of occurrence were used as background for two species. The null distributions were reconstructed from 999 random sampling replications and validated by *env.D* and *env.I*, which are improved indices of similarity between niches, with multiple corrections using false discovery rate. To investigate the association between bio19 and snowfall, the Mean Temperature of Coldest Quarter (bio11) of occurrence locations was obtained from the current environments using raster package (version 3.5‐21) and tested using the Wilcoxon rank sum test with the coin package (version 1.4‐2) in R.

### 2.3 DNA extraction and sequencing

Total genomic DNA was extracted using hexadecyltrimethylammonium bromide (CTAB) method (Murray & Thompson, 1980), with minor modification. To acquire haplotypes of cpDNA, primers for *rbcL* (Kress et al., 2009) and *psbA*‐*trnH* intergenic spacer (Sang et al., 1997) were used for 1st PCR. These primers were preceded by one to three random bases (N, NN, or NNN). For the second PCR, complementary sequences for the binding sites of the Illumina sequencing flow cell and barcode sequences for each sample were added to the first PCR products. Both PCRs were performed using PrimeSTAR HS DNA Polymerase (Takara). These templates were pooled and sequenced using an Illumina MiSeq sequencer (Illumina) with Reagent Kit v2 (500 cycle, paired‐end).

To acquire genome‐wide SNPs as nuclear markers, multiplexed inter‐simple sequence repeats genotyping by sequencing (MIG‐seq) was performed (Suyama et al., 2022). MIG‐seq is a reduced‐representation DNA sequencing method that uses a high‐throughput sequencing platform to detect putatively neutral genome‐wide SNPs adjacent to microsatellite regions. It is used for low‐quality DNA, such as museum specimens, because of the PCR amplification‐based approach (Strijk et al., 2020). The MIG‐seq library was sequenced using an Illumina MiSeq sequencer with a Reagent Kit v3 (150 cycle, paired‐end).

### 2.4 Genotyping

Reads obtained from cpDNA sequencing were analyzed according to Claident protocol (Tanabe & Toju, 2013) using the recommended parameters (https://www.claident.org). Raw data obtained from the sequencer were called in fastq format using bcl2fastq v2 (Illumina), and the reads were demultiplexed. Because of different insert sizes, paired‐end reads of *psbA*‐*trnH* were merged at overlapping regions, and *rbcL* reads were concatenated. Low‐quality or noisy sequence were filtered out, and passing sequences were clustered by 100% identity. The most frequently acquired reads of each sample were obtained as representative sequences. The representative sequences of all samples accounted for more than 50% of the obtained sequences, and the genera were confirmed using the Basic Local Alignment Search Tool (BLAST; https://blast.ncbi.nlm.nih.gov/Blast.cgi). These treatments were applied to reduce contaminations. The representative sequences were aligned using MAFFT (Katoh & Standley, 2013), and 5‐bp around gaps were trimmed for proper alignment. Lastly, the representative sequences of each sample were concatenated.

MIG‐seq reads were trimmed by removing adapter sequences and low‐quality reads using Trimmomatic version 0.39 (Bolger et al., 2014). The filtered reads were genotyped using stacks version 2.4 (Rochette et al., 2019) for de novo assembly. The parameter of minimum depth of coverage required to classify reads as “stack” was set to three (*m* = 3). The number of mismatches allowed between stacks and to align secondary reads were set to one and two, respectively (*M* = 1, *n* = 1, *N* = 2). These mismatch parameters are relatively low to avoid misassembly of paralogs since *S. renifolius* is tetraploid. This reduces the number of SNPs but is more conservative. The stacks were genotyped as SNPs if they were typed as at least 70% of the target species, minor alleles were counted to more than two, and observed heterozygosity was less than 60% using the “populations” command. The parameter settings were increased to obtain more SNPs (*M* = 2, *n* = 2, *N* = 4) yielded qualitatively similar results.

### 2.5 Phylogeographic and population structure analysis

Public sequence data were downloaded from the National Center for Biotechnology Information (NCBI) database (https://www.ncbi.nlm.nih.gov), including the three *S. foetidus rbcL* and *psbA*‐*trnH* sequences of SCBI‐SIGEO_13_0008, SCBI‐SIGEO_13_0009, and AD5JS54; the two *S. nipponicus* chloroplast genomes MK158079.1, MK341566.1 (Kim et al., 2019); and one *S. renifolius* chloroplast genome, KY039276.1 (Choi et al., 2017). The haplotype network constructed using integer neighbor‐joining methods in PopART was visualized (Leigh & Bryant, 2015). Sampling locations were mapped using the worldHires and mapdata packages (version 2.3.0) in R.

Populations with four or more amplified individuals were used for nuclear DNA analysis. A total of 135 individuals from 15 populations of *S. renifolius* (mean of 9.0 individuals per population, 5–24) and 92 individuals from eight populations of *S. nipponicus* (mean of 11.5 individuals per population, 6–24) were analyzed (Table S1). The rate of the polymorphic loci, nucleotide diversity (*π*), observed heterozygosity (*H*_o_), and the number of private alleles were calculated by the “populations” command in Stacks as summary statistics of genetic diversity for each population of both species. Phylogenetic relationships among individuals were determined by maximum likelihood estimation using RAxML version 8.2.12 (Stamatakis, 2014) with GTRGAMMA model and visualized using ggtree (Yu et al., 2017). The population structure of each species was determined using ADMIXTURE version 1.3.0 (Alexander & Lange, 2011). The numbers of clusters were tested from 1 to 15, and the other parameters were set by default values. The best *K* values were selected based on cross‐validation error.

## 3 RESULTS

### 3.1 Comparison of potential distribution and response to past climate change

**FIGURE 2:**
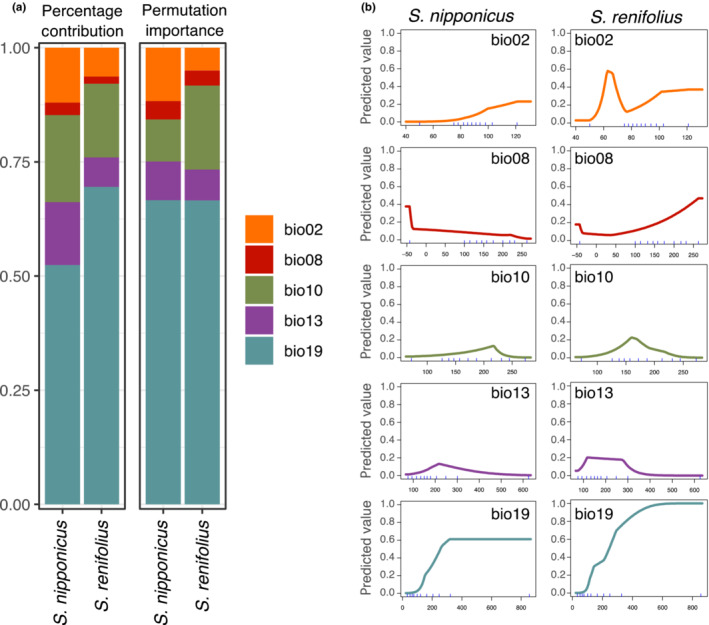
Effects of each environmental valuable to the Maxent model. (a) Percent contribution (left) and permutation importance (right) of five environmental variables in *Symplocarpus nipponicus* and *Symplocarpus renifolius*. bio02, mean diurnal range; bio08, mean temperature of wettest quarter; bio10, mean temperature of warmest quarter; bio13, precipitation of wettest month; bio19, precipitation of coldest quarter. (b) Response curves of the five environmental variables. The predicted probability of presence fluctuates as each environmental variable has varied.

To investigate the relationship between plant thermogenesis and cold adaptation, the potential distributions of thermogenic *S. renifolius* and non/slightly thermogenic *S. nipponicus* were determined and compared using Maxent. Maxent models for both species showed high accuracy in predicting the observed distributions, with high AUC values (>0.9) for the best parameters (Figure 2; Figure S2). Of the five environmental variables (bio02, 08, 10, 13, and 19), precipitation of coldest quarter (bio19) contributed the most to both percent contribution and permutation importance (Figure 2a) in both *Symplocarpus* species. The effects of each environmental variable on the Maxent prediction were shown as response curves (Figure 2b). Higher precipitation of the coldest quarter (bio19), which is the largest contributor for both species, increased the probability of presence in both species. The predicted value for bio19 in *S. nipponicus* reached a plateau around the middle level, whereas that in *S. renifolius* reached a plateau at the maximal level. The mean temperature of the coldest quarter (bio11) at the occurring points was compared between species to determine whether precipitation during winter contained snow in the area where both species were distributed (Figure S3). Bio11 was less than or equal to 0°C in both species, and the area covered by *S. renifolius* was colder than that of *S. nipponicus* (Wilcoxon rank sum test, *p* = .015). Whether the niches between *S. nipponicus* and *S. renifolius* are more different from those predicted at random was tested using the niche identity test and symmetric background test. The empirical niche overlap between *S. renifolius* and *S. nipponicus* was significantly lower than expected from random in both tests of niche identity (*env.D*: *p* = .001, *env.I*: *p* = .001) and symmetric background (*env.D*: *p* = .01, *env.I*: *p* = .01).

**FIGURE 3:**
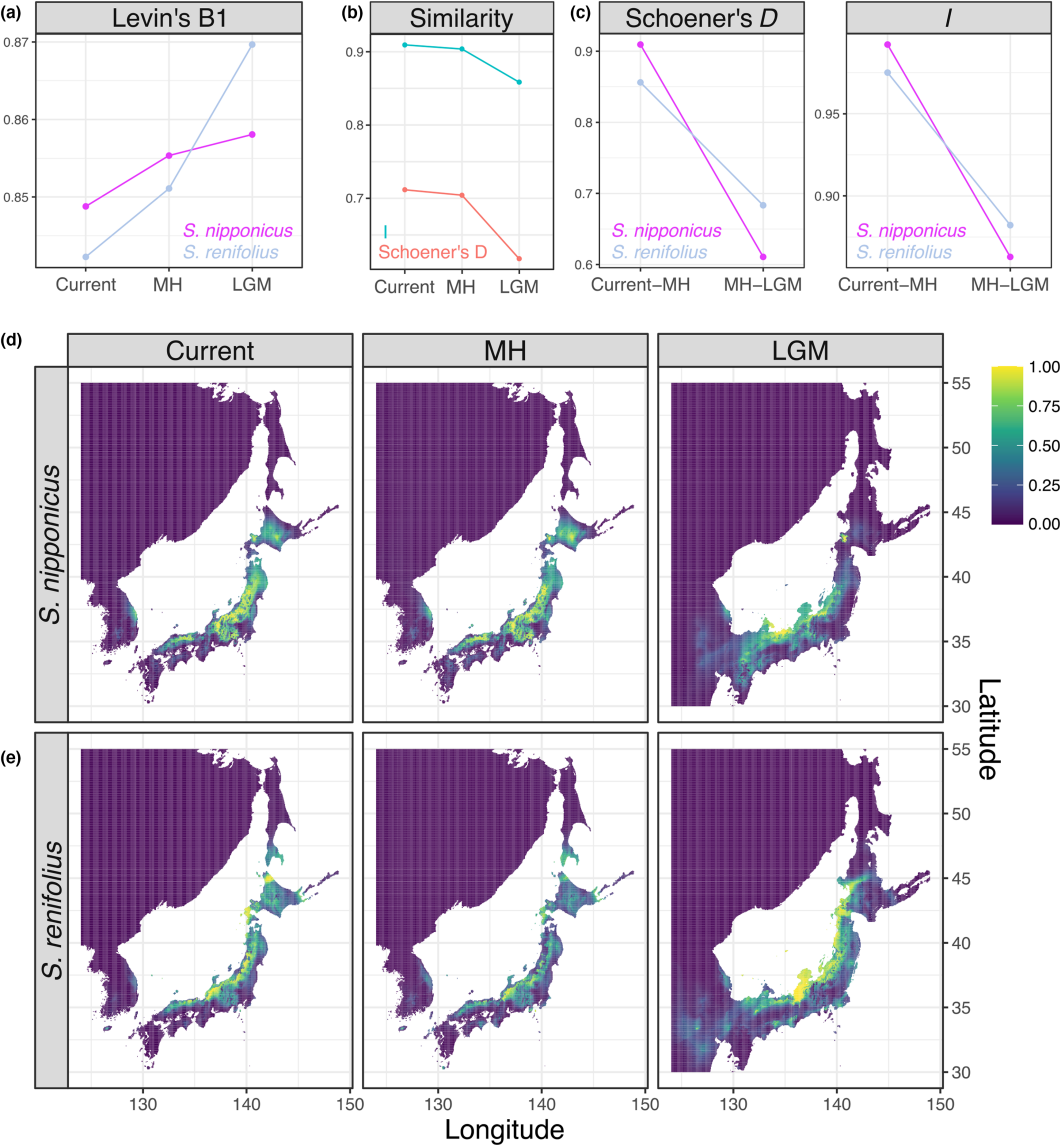
Comparison of potential distributions predicted by Maxent. (a) Levin's niche breadth. (b) Niche similarity between two species by Schoener's *D* and *I*. (c) Niche similarity across ages by Schoener's *D* and *I*. Visualization of occurrence probability around Northeast Asia in (d) *Symplocarpus nipponicus* and (e) *Symplocarpus renifolius* using (left) current climatic conditions, (middle) the Mid‐Holocene, and (right) the Last Glacial Maximum. Color bar indicates the intensity of occurrence probability.

To assess the response to past climate change, the past distribution of both species in the Mid‐Holocene (MH, approximately 6000 years ago) and Last Glacial Maximum (LGM, about 22,000 years ago) was estimated and compared with the current distribution (Figure 3). Compared with the current environment, the environment around Japan in the MH is hotter while that in the LGM is colder. Comparing Levin's niche breadth among climate models with less extrapolation, *S. renifolius* distributed a wide breadth of niche region in the LGM and decrease in the MH, whereas *S. nipponicus* kept the breadth of distribution since the LGM (Figure 3a). The similarity of predicted distribution between two species in the LGM was lower than that in the current and MH in both indexes (Figure 3b). Comparing the similarity between the ages, the similarity between the MH and the LGM in *S. nipponicus* was lower than that in *S. renifolius* in both indexes (Figure 3c), suggesting different distribution shift between species from the LGM to the current environment. These notions were further confirmed by the visualization (Figure 3d,e). The distribution of *S. nipponicus* in the MH was similar to the current distribution, while the distribution with higher probability in the LGM was narrower and more sporadic (Figure 3d). In contrast, when compared to the current distribution, the distribution of *S. renifolius* in the MH was narrower and more sporadic, with a lower probability, while that in the LGM was broader (Figure 3e; Figure S4). Based on the Maxent model, the potential distribution of *S. renifolius* was widely expanded to the colder and snowier northern region than that of *S. nipponicus* in the Japanese islands and Sakhalin, which roughly corresponds to its current occurrence (Figures 1 and 3). Overall, these results showed that the niche of *S. nipponicus* has kept the breadth and migrated since the LGM, the niche of *S. renifolius* has decreased since the LGM, and the snowfall in the coldest quarter contributed the most to the distribution pattern of the two species.

### 3.2 Phylogenetic and geographic relationship of cpDNA in the genus *Symplocarpus*

**FIGURE 4:**
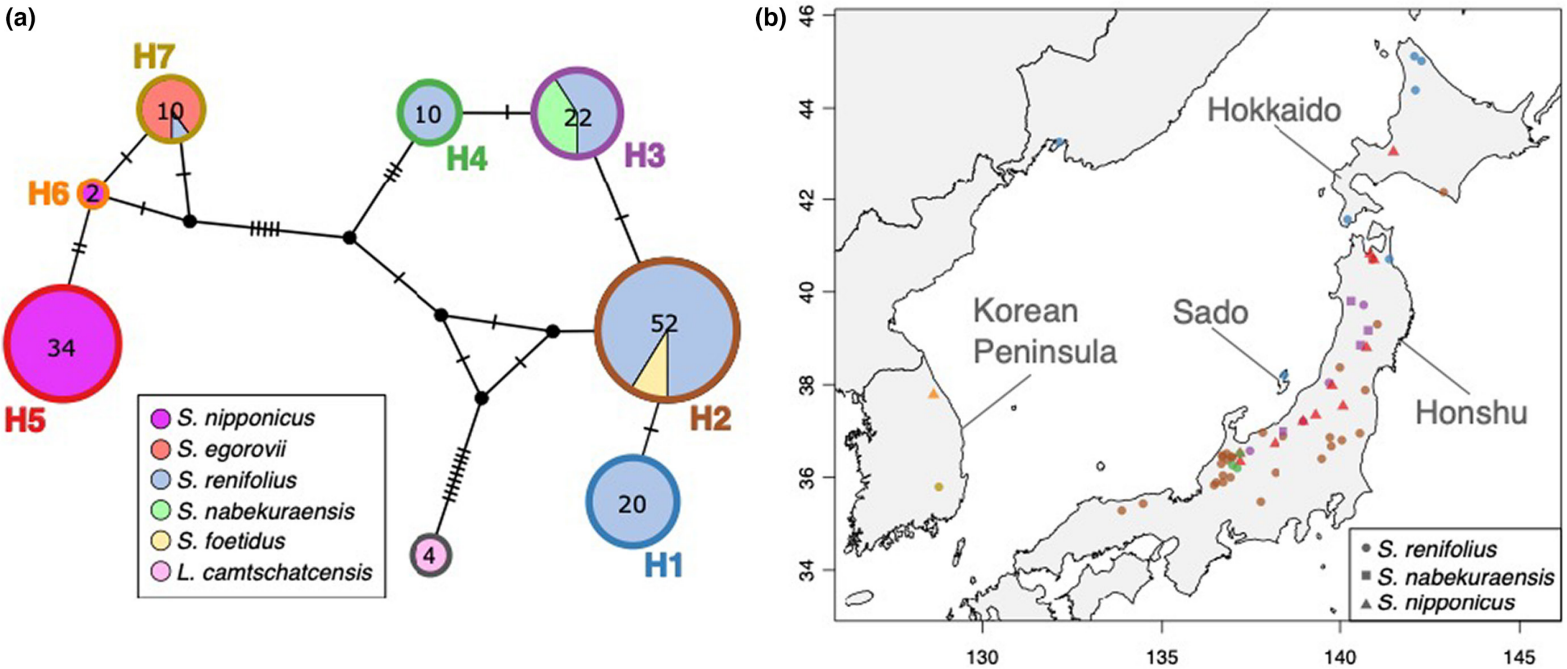
Phylogeographic analysis of cpDNA for the *Symplocarpus* species in Northeast Asia. (a) Haplotype network of five *Symplocarpus* species and *L. camtschatcensis*. Colors of pie charts indicate species and colors of circumference of pie charts indicate haplotypes (H1–H7). Numbers in pie charts indicate sample size, and the size of each circle is proportional to haplotype frequency. Bars indicate the number of point mutations between two haplotypes, and small black circles indicate undetected haplotypes. (b) Geographic distribution of chloroplast haplotype for three skunk cabbage species. Sampled sites are indicated as follows: circle, *Symplocarpus renifolius*; square, *Symplocarpus nabekuraensis*; and triangle, *Symplocarpus nipponicus*. The colors of each map symbol indicate haplotypes (H1–H7) and are consistent with the pie chart outline color in (a).

To examine whether the different distribution patterns of the two *Symplocarpus* species during the past age have affected the genetic diversity or population structure in the current age, the phylogenetic and geographic relationships were analyzed using cpDNA. Partial sequencing of the *rbcL* and *psbA*‐*trnH* intergenic spacer on cpDNA (725 bp) for 162 samples of five *Symplocarpus* species and *L. camtschatcensis* (outgroup) showed different genetic diversities across Japan for non/slightly thermogenic *S. nipponicus* and thermogenic *S. renifolius*. Only one haplotype of *S. nipponicus* was observed in Japan, whereas four haplotypes of *S. renifolius* sharing the same haplotype within a population were observed (H1: northern haplotype from Sakhalin to Hokkaido and Sado island, H2: broadly distributed haplotype in Honshu and southern Hokkaido, H3: mountain haplotype in the Sea of Japan side in northern Honshu, and H4: local haplotype in the partial region) (Figure 4). The most genetically distant haplotypes were the northern (H1) and local (H4) haplotype with three SNPs, despite comparison of short sequencing regions. The haplotype of *S. nipponicus* in Korea (H6) was different from that of *S. nipponicus* in Japan (H5) by two SNPs. *S. nabekuraensis* and *S. foetidus* were included in haplotypes (H3) and (H2), respectively.

### 3.3 Phylogenetic and geographic relationship of genome‐wide SNPs by MIG‐seq

**FIGURE 5:**
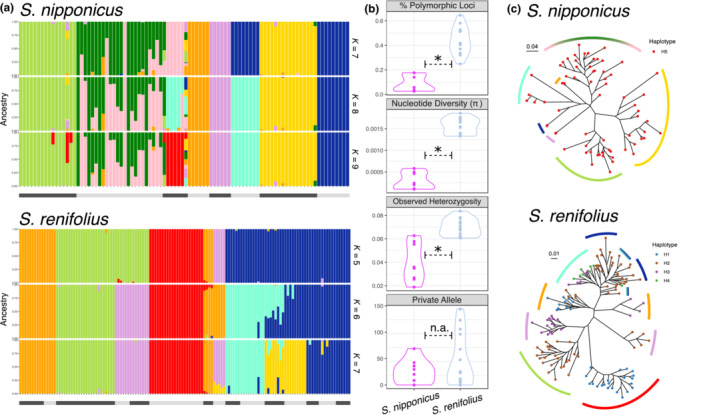
Phylogeographic analyses of genome‐wide SNPs for *Symplocarpus nipponicus* (541 SNPs from 92 individuals) and *Symplocarpus renifolius* (2434 SNPs from 135 individuals). (a) Population structure in Japan using ADMIXTURE. The proportion of ancestry for each individual in *K* (the number of clusters) = 7, 8, or 9 for *S. nipponicus*, and *K* = 5, 6, or 7 for *S. renifolius* is shown. The best *K* values based on cross‐validation error are *K* = 8 in *S. nipponicus* and *K* = 6 in *S. renifolius* (Figure S5). Black and gray bars at the bottom indicate populations. (b) Comparison of genetic diversity between *S. renifolius* and *S. nipponicus* (Table S1). Asterisk indicates a significant difference between species using Welche's t‐test with Benjamini and Hochberg correction. (c) Phylogenetic tree using RAxML. Dot colors indicate haplotype of cpDNA (Figure 4) and colored bars around the trees indicate population structure of best *K* in (a).

To perform a detailed comparison of phylogenetic relationships and population structure within the populations of *S. nipponicus* and *S. renifolius* by nuclear genome sequencing, MIG‐seq was performed using a high‐throughput sequencer (Figure 5). MIG‐seq is a powerful tool for genome‐wide identification of SNPs by sequencing the region between microsatellites. The evolutionary timescale in nuclear genome is largely different from that in the chloroplast genome, and generally, evolutionary processes in the nuclear genome are more rapid than those in the chloroplast genome (Drouin et al., 2008). A total of 2434 SNPs in 119,116 bp of 135 *S. renifolius* individuals and 541 SNPs in 141,018 bp of 94 *S. nipponicus* individuals were obtained using MIG‐seq. In both species, population structure analysis using ADMIXTURE showed a more distinct differentiation among populations than cpDNA analysis. A clearly diverged structure for each population was found in both *S. nipponicus* and *S. renifolius*, as the best number of clusters explained by cross‐validation error was eight in nine populations of *S. nipponicus* and six in 15 populations of *S. renifolius* (Figure 5a; Figure S5). The percentage of polymorphic loci, nucleotide diversity, and observed heterozygosity in the nuclear genome of *S. renifolius* was significantly higher than those in *S. nipponicus* (*p* < .005, Welch's *t*‐test with Benjamini and Hochberg correction (Benjamini & Hochberg, 1995) for multiple testing), but the number of the private allele was not significant (*p* = .208) (Figure 5b; Table S1). In *S. renifolius* with higher genetic diversity, the phylogenetic relationship was topologically inconsistent with the haplotype network analyses using cpDNA (Figures 4a and 5c).

## 4 DISCUSSION

### 4.1 Contribution of floral thermogenesis to niche divergence, cold adaptation, and pollination ecology

This study shows that the species exhibiting higher thermogenesis also lives in colder climates. The current distributions between species were significantly different from those predicted from random using ENM comparison, suggesting niche divergence. The different responses of the distributions between species were illustrated by the past fluctuated environment of the MH, which was hotter than the current environment, and the LGM, which was colder than the current environment. *S. renifolius* expanded its distribution in the LGM and decreased during the MH, whereas *S. nipponicus* kept the breadth of distribution since the LGM and migrated to the northern region in the MH (Figure 3). As a result, the current distribution of the two species overlaps more than the distribution in the LGM.

The different distribution response in the past between species has been supported by higher genetic diversity in *S. renifolius* (Figure 5). These results show the history of niche divergence between *S. renifolius* and *S. nipponicus*; that the species with increased heat‐producing ability lives in colder climates, and the niche divergence may imply that floral thermogenesis contributes to cold adaptation in *Symplocarpus*. *S. renifolius* is able to maintain the spadices at approximately 20°C during the thermogenic stage (Ito‐Inaba et al., 2009). The remarkably high heat‐producing ability of this species contributes to the relief of cold stress or promotes blooming by activating cellular metabolism during the snowmelt season. The distributions of non/slightly thermogenic *S. nipponicus* did not decrease in the MH, although it was also affected by precipitation in winter. In this species, blooming in early summer may prevent the consumption of enormous amounts of energy by thermogenesis. However, it remains possible that factors other than thermogenesis, such as size difference (Figure 1), might have influenced the distribution patterns and evolutionary processes of *S. renifolius* and *S. nipponicus*. In future studies, it will be of great interest to understand how multiple factors drive the evolution of these species.

Based on ENM by Maxent, the distributions of thermogenic *S. renifolius* and non/slightly thermogenic *S. nipponicus* were determined by precipitation in winter, which is the amount of snowfall, and the predicted probability of presence showed different response (Figure 2). Niches determination of the species by environmental variables of seasonal precipitation (bio19 and bio13), not temperature (bio2, bio8, and bio10), would be associated with seasonality of their life histories. *S. renifolius* is flowering before leafing in the spring, while *S. nipponicus* is flowering after leafing in the spring, and ground parts of both species wither in the fall (Ito‐Inaba et al., 2016; Otsuka, 2002). Disappearance of ground parts in the warmest and coldest seasons would be less affected by ambient temperature. On the contrary, the amount of snowfall may affect the soil environment during the leafing season, as the snow melts during spring and summer. Preference for damp ground will cause species to determine their niches according to precipitation. Although the causal association between snowfall and thermogenesis is not directly shown, higher thermogenic species cover deeper‐snow areas, while non/slightly thermogenic species are restricted by factors associated with snowfall environments.

If floral thermogenesis contributes to cold adaptation in the thermogenic *Symplocarpus*, genetic vestiges remain, such that the genetic diversity of thermogenic species is higher than that of non/slightly thermogenic species because of the avoidance of distribution reduction in an ice age. Comparative analysis between thermogenic *S. renifolius* and non/slightly thermogenic *S. nipponicus* has revealed the different genetic divergence in cpDNA and nuclear DNA, as well as the topological discordance of phylogenetic relationship between chloroplast and nuclear genome in *S. renifolius* and fragmentary population genetic structure in nuclear DNA (Figures 4 and 5). Topological discordance between organelle and nuclear genome has occasionally been observed in animals (Ford et al., 2019; Singhal & Moritz, 2012) and plants (Hirota et al., 2021; Petit et al., 2005; Xu et al., 2021). This may be due to the different patterns of gene flow between maternally inherited organelles and biparentally inherited nuclei, and sex‐biased dispersal between seed and pollen. Although seeds usually fall into muddy substrates or are carried off by animals and floods, almost all seeds of both species are consumed by rodents in Japan (Otsuka & Kitano, 2003) and the range of seed dispersal by rodents has been estimated to be approximately 10 m in *S. renifolius* (Abe et al., 2006; Wada & Uemura, 1994). In contrast, pollen of *Symplocarpus* is dispersed by flies (Chartier et al., 2014), and a large variety of Coleoptera and Diptera are attracted to thermogenic *S. foetidus* in Canada (Barriault et al., 2021). Pollen dispersal by various pollinators could cause large gene flow in the nuclear genome among populations of *S. renifolius*, although seed dispersal is rarely different between thermogenic and non/slightly thermogenic species. These genetic vestiges in *S. renifolius* suggest the contribution of odor dispersal and attraction of pollinators by floral thermogenesis to topological discordance and genetic diversity.

Global warming by climate change in the future will influence the distributions in *Symplocarpus* explained by the amount of snowfall. According to the response curves of precipitation of the coldest quarter (bio19), especially in the deep‐snow area, less snowfall decreases the distribution in thermogenic *S. renifolius*. At the same time, it may have a relatively small effect on the distribution in non/slightly thermogenic *S. nipponicus* (Figure 2). In addition to the inference from ENM, temperature elevation may eliminate the effectiveness of thermogenesis and require more costs. The results are that thermogenic species will not be able to attract pollinators and provide benefits to them in early spring. This suggestion applies to thermogenic species that prefer cold environments, although climatic change will also affect to other thermogenic plants according to role of heat in each species.

Moreover, a comparison between thermogenic and non/slightly thermogenic species revealed different response to the amount of snowfall and different historical distributions using Maxent. It also revealed different ranges of pollen dispersal from phylogeographic discordance between cpDNA and genome‐wide SNPs. Our findings suggest that floral thermogenesis is a consequence of adaptive evolution due to cold environment and pollination, but it remains a possibility that the results are caused by the history of distribution formation after speciation, instead of adaptation. The possibility that phylogeographic discordance is caused by positive selection could not be investigated in this study because of limited genetic information. Closely related species available for verification are *L. camtschatcensis*, which is non‐thermogenic and broadly distributed in Japan and Russia, and *S. nabekuraensis*, which is thermogenic and locally distributed in high‐altitude areas in Japan, but an endangered species. To elucidate the adaptive signature of thermogenesis in *Symplocarpus*, genome sequencing, understanding thermogenic mechanisms, and detecting the natural selection of thermogenic genes are necessary.

### 4.2 Phylogeography and speciation process in *Symplocarpus*

The phylogenetic relationship between cpDNA and the nuclear genome of *Symplocarpus* was consistent with previous studies, except for the outgroup *L. camtschatcensis* (Kitano et al., 2005; Lee et al., 2019; Nie et al., 2006). Two major lineages were found in *Symplocarpus*: one lineage included *S. renifolius*, *S. foetidus*, and *S. nabekuraensis*, and the other included *S. nipponicus* and *S. egorovii*. Two of the four haplotypes of *S. renifolius* were closely related to other thermogenic species: *S. nabekuraensis* and *S. foetidus*. The haplotype of *S. nabekuraensis* was consistent with the mountain haplotype in the Sea of Japan side in northern Honshu (H3). This species is distributed in high‐altitude and deep‐snow areas in the Sea of Japan, like H3 of *S. renifolius* (Otsuka, 2002). *S. nabekuraensis* might have been derived from H3 of *S. renifolius* and resulted from geographical isolation at refugia or adaptation to higher altitude areas. The haplotype of *S. foetidus* was consistent with the broadly distributed haplotypes in Honshu and southern Hokkaido (H2). *S. foetidus* is found in eastern North America and its distribution does not overlap with that of *S. renifolius*. When the H2 of *S. renifolius* spreads throughout northern Japan, it might have expanded to North America across the Pacific Ocean and formed distinct species through geographical isolation. Speciation and distribution expansion in *Symplocarpus* were observed by phylogeographic analysis and sampling covering a wide area in Japan.

*Symplocarpus nipponicus* in Japan had one haplotype (H5), which differed from the haplotypes of *S. nipponicus* in Korea (H6) and *S. egorovii* (H7), a new species described in 2005 (Pavlova & Nechaev, 2005). The H7 includes all *S. egorovii* and one *S. renifolius*, registered as chloroplast genome of *S. renifolius* in Korea (Choi et al., 2017) and distinguished from the haplotype of *S. renifolius* in Japan (H1–H4). The *S. renifolius* sample of H7 is a living collection in South Korea. *S. renifolius* in Korea is more genetically related to *S. egorovii* and *S. nipponicus* than *S. renifolius* in Japan (Lee et al., 2019), so the chloroplast genome identified as *S. renifolius* in Korea might be *S. egorovii* and *S. koreanus* recently described from Russia and Korea, respectively (Lee et al., 2021; Pavlova & Nechaev, 2005). According to the habitat ranges estimated by occurrence data in Japan using Maxent model, the potential current distribution of *S. renifolius* in South Korea seems to have a lower probability than that of *S. nipponicus*. Phylogeographic analysis using cpDNA will reveal the speciation process across this clade, including *S. koreanus*, in Northeast Asia.

## AUTHOR CONTRIBUTIONS

**Mitsuhiko P. Sato:** Conceptualization (lead); data curation (lead); formal analysis (lead); funding acquisition (equal); investigation (lead); methodology (lead); project administration (lead); validation (lead); visualization (lead); writing – original draft (lead). **Ayumi Matsuo:** Investigation (lead); writing – review and editing (supporting). **Koichi Otsuka:** Data curation (supporting); resources (equal); writing – review and editing (supporting). **Kohei Takenaka Takano:** Formal analysis (supporting); methodology (supporting); resources (supporting); software (supporting); supervision (supporting); writing – review and editing (supporting). **Masayuki Maki:** Resources (equal). **Kunihiro Okano:** Methodology (supporting); software (supporting); writing – review and editing (supporting). **Yoshihisa Suyama:** Funding acquisition (supporting). **Yasuko Ito‐Inaba:** Conceptualization (supporting); funding acquisition (equal); supervision (lead); writing – review and editing (lead).

## Supporting information


Figures S1–S5

Table S1

## Data Availability

Raw sequencing files have been deposited in the DDBJ PRJDB14116. The outline of the R scripts has been uploaded on GitHub (https://github.com/MitsuhikoP/SymplocarpusENM.git).

## References

[ece310319-bib-0001] Abe, H. , Matsuki, R. , Ueno, S. , Nashimoto, M. , & Hasegawa, M. (2006). Dispersal of *Camellia japonica* seeds by *Apodemus speciosus* revealed by maternity analysis of plants and behavioral observation of animal vectors. Ecological Research, 21(5), 732–740.

[ece310319-bib-0002] Alexander, D. H. , & Lange, K. (2011). Enhancements to the ADMIXTURE algorithm for individual ancestry estimation. BMC Bioinformatics, 12, 246. 10.1186/1471-2105-12-246 21682921 PMC3146885

[ece310319-bib-0003] Angioy, A. M. , Stensmyr, M. C. , Urru, I. , Puliafito, M. , Collu, I. , & Hansson, B. S. (2004). Function of the heater: The dead horse arum revisited. Proceedings of the Royal Society B: Biological Sciences, 271(Suppl 3), S13–S15. 10.1098/rsbl.2003.0111 PMC1809992 15101405

[ece310319-bib-0004] Barriault, I. , Barabé, D. , Cloutier, L. , Pellerin, S. , & Gibernau, M. (2021). Pollination ecology of *Symplocarpus foetidus* (Araceae) in a seasonally flooded bog in Québec, Canada. Botany Letters, 168(3), 373–383.

[ece310319-bib-0005] Benjamini, Y. , & Hochberg, Y. (1995). Controlling the false discovery rate: A practical and powerful approach to multiple testing. Journal of the Royal Statistical Society: Series B (Statistical Methodology), 57(1), 289–300.

[ece310319-bib-0006] Blair, M. E. , Sterling, E. J. , Dusch, M. , Raxworthy, C. J. , & Pearson, R. G. (2013). Ecological divergence and speciation between lemur (*Eulemur*) sister species in Madagascar. Journal of Evolutionary Biology, 26(8), 1790–1801. 23865477 10.1111/jeb.12179

[ece310319-bib-0007] Bolger, A. M. , Lohse, M. , & Usadel, B. (2014). Trimmomatic: A flexible trimmer for Illumina sequence data. Bioinformatics, 30(15), 2114–2120. 24695404 10.1093/bioinformatics/btu170 PMC4103590

[ece310319-bib-0008] Breiman, L. (2001). Random forests. Machine Learning, 45, 5–32.

[ece310319-bib-0009] Chartier, M. , Gibernau, M. , & Renner, S. S. (2014). The evolution of pollinator–plant interaction types in the Araceae. Evolution, 68(5), 1533–1543. 24274161 10.1111/evo.12318

[ece310319-bib-0010] Choi, K. S. , Park, K. T. , & Park, S. J. (2017). The chloroplast genome of *Symplocarpus renifolius*: A comparison of chloroplast genome structure in Araceae. Genes, 8(11), 324. 10.3390/genes8110324 29144427 PMC5704237

[ece310319-bib-0011] Drake, J. M. , Randin, C. , & Guisan, A. (2006). Modelling ecological niches with support vector machines. The Journal of Applied Ecology, 43(3), 424–432.

[ece310319-bib-0012] Drouin, G. , Daoud, H. , & Xia, J. (2008). Relative rates of synonymous substitutions in the mitochondrial, chloroplast and nuclear genomes of seed plants. Molecular Phylogenetics and Evolution, 49(3), 827–831. 18838124 10.1016/j.ympev.2008.09.009

[ece310319-bib-0013] Ford, A. G. P. , Bullen, T. R. , Pang, L. , Genner, M. J. , Bills, R. , Flouri, T. , Ngatunga, B. P. , Rüber, L. , Schliewen, U. K. , Seehausen, O. , Shechonge, A. , Stiassny, M. L. J. , Turner, G. F. , & Day, J. J. (2019). Molecular phylogeny of *Oreochromis* (Cichlidae: Oreochromini) reveals mito‐nuclear discordance and multiple colonisation of adverse aquatic environments. Molecular Phylogenetics and Evolution, 136, 215–226. 30974200 10.1016/j.ympev.2019.04.008

[ece310319-bib-0014] Fuchs, A. J. , Gilbert, C. C. , & Kamilar, J. M. (2018). Ecological niche modeling of the genus *Papio* . American Journal of Physical Anthropology, 166(4), 812–823. 29607482 10.1002/ajpa.23470

[ece310319-bib-0015] Gonçalves‐Souza, P. , Schlindwein, C. , Dötterl, S. , & Paiva, E. A. S. (2017). Unveiling the osmophores of *Philodendron adamantinum* (Araceae) as a means to understanding interactions with pollinators. Annals of Botany, 119(4), 533–543. 28065928 10.1093/aob/mcw236 PMC5458670

[ece310319-bib-0016] Guisan, A. , Edwards, T. C., Jr. , & Trevor, H. (2002). Generalized linear and generalized additive models in studies of species distributions: Setting the scene. Ecological Modeling, 157, 89–100.

[ece310319-bib-0017] Hijmans, R. J. , Cameron, S. E. , Parra, J. L. , Jones, P. G. , & Jarvis, A. (2005). Very high resolution interpolated climate surfaces for global land areas. International Journal of Climatology, 25(15), 1965–1978.

[ece310319-bib-0018] Hirota, S. K. , Yasumoto, A. A. , Nitta, K. , Tagane, M. , Miki, N. , Suyama, Y. , & Yahara, T. (2021). Evolutionary history of *Hemerocallis* in Japan inferred from chloroplast and nuclear phylogenies and levels of interspecific gene flow. Molecular Phylogenetics and Evolution, 164, 107264. 34273506 10.1016/j.ympev.2021.107264

[ece310319-bib-0019] Ito‐Inaba, Y. , Hida, Y. , Ichikawa, M. , Kato, Y. , & Yamashita, T. (2008). Characterization of the plant uncoupling protein, SrUCPA, expressed in spadix mitochondria of the thermogenic skunk cabbage. Journal of Experimental Botany, 59(4), 995–1005. 18308738 10.1093/jxb/ern024

[ece310319-bib-0020] Ito‐Inaba, Y. , Hida, Y. , Mori, H. , & Inaba, T. (2008). Molecular identity of uncoupling proteins in thermogenic skunk cabbage. Plant & Cell Physiology, 49(12), 1911–1916. 18974196 10.1093/pcp/pcn161

[ece310319-bib-0021] Ito‐Inaba, Y. , Masuko‐Suzuki, H. , Maekawa, H. , Watanabe, M. , & Inaba, T. (2016). Characterization of two PEBP genes, SrFT and SrMFT, in thermogenic skunk cabbage (*Symplocarpus renifolius*). Scientific Reports, 6, 1–14. 27389636 10.1038/srep29440 PMC4937424

[ece310319-bib-0022] Ito‐Inaba, Y. , Sato, M. , Masuko, H. , Hida, Y. , Toyooka, K. , Watanabe, M. , & Inaba, T. (2009). Developmental changes and organelle biogenesis in the reproductive organs of thermogenic skunk cabbage (*Symplocarpus renifolius*). Journal of Experimental Botany, 60(13), 3909–3922. 19640927 10.1093/jxb/erp226 PMC2736897

[ece310319-bib-0023] Ito‐Inaba, Y. , Sato, M. , Sato, M. P. , Kurayama, Y. , Yamamoto, H. , Ohata, M. , Ogura, Y. , Hayashi, T. , Toyooka, K. , & Inaba, T. (2019). Alternative oxidase capacity of mitochondria in microsporophylls may function in cycad thermogenesis. Plant Physiology, 180(2), 743–756. 30918084 10.1104/pp.19.00150 PMC6548267

[ece310319-bib-0024] Katoh, K. , & Standley, D. M. (2013). MAFFT multiple sequence alignment software version 7: Improvements in performance and usability. Molecular Biology and Evolution, 30(4), 772–780. 23329690 10.1093/molbev/mst010 PMC3603318

[ece310319-bib-0025] Kim, S.‐H. , Cho, M.‐S. , Li, P. , & Kim, S.‐C. (2018). Phylogeography and ecological niche modeling reveal reduced genetic diversity and colonization patterns of skunk cabbage (*Symplocarpus foetidus*; Araceae) from glacial refugia in Eastern North America. Frontiers in Plant Science, 9, 1–19. 29872442 10.3389/fpls.2018.00648 PMC5972301

[ece310319-bib-0026] Kim, S. H. , Yang, J. Y. , Park, J. , Yamada, T. , Maki, M. , & Kim, S. C. (2019). Comparison of whole plastome sequences between thermogenic skunk cabbage *Symplocarpus renifolius* and nonthermogenic *S. nipponicus* (Orontioideae; Araceae) in East Asia. International Journal of Molecular Sciences, 20(19), 4678. 10.3390/ijms20194678 31547213 PMC6801674

[ece310319-bib-0027] Kitano, S. , Otsuka, K. , Uesugi, R. , & Goka, K. (2005). Molecular phylogenetic analysis of the genus *Symplocarpus* (Araceae) from Japan based on chloroplast DNA sequences. Journal of Japanese Botany, 80, 334–339.

[ece310319-bib-0028] Knutson, R. M. (1974). Heat production and temperature regulation in eastern skunk cabbage. Science, 186(10), 746–748. 4417289 10.1126/science.186.4165.746

[ece310319-bib-0029] Kress, W. J. , Erickson, D. L. , Jones, F. A. , Swenson, N. G. , Perez, R. , Sanjur, O. , & Bermingham, E. (2009). Plant DNA barcodes and a community phylogeny of a tropical forest dynamics plot in Panama. Proceedings of the National Academy of Sciences of the United States of America, 106(44), 18621–18626. 19841276 10.1073/pnas.0909820106 PMC2763884

[ece310319-bib-0030] Lee, J. S. , Kim, S.‐H. , Kim, Y. , Kwon, Y. , Yang, J. , Cho, M.‐S. , Kim, H.‐B. , Lee, S. , Maki, M. , & Kim, S.‐C. (2021). *Symplocarpus koreanus* (Araceae; Orontioideae), a new species based on morphological and molecular data. Korean Journal of Plant Taxonomy, 51(1), 1–9.

[ece310319-bib-0031] Lee, J. S. , Kim, S. H. , Lee, S. , Maki, M. , Otsuka, K. , Kozhevnikov, A. E. , Kozhevnikova, Z. V. , Wen, J. , & Kim, S. C. (2019). New insights into the phylogeny and biogeography of subfamily Orontioideae (Araceae). Journal of Systematics and Evolution, 57(6), 616–632.

[ece310319-bib-0032] Leigh, J. W. , & Bryant, D. (2015). POPART: Full‐feature software for haplotype network construction. Methods in Ecology and Evolution, 6(9), 1110–1116.

[ece310319-bib-0033] Levins, R. (1968). Evolution in changing environments. Princeton University Press.

[ece310319-bib-0034] Li, J.‐K. , & Huang, S.‐Q. (2009). Flower thermoregulation facilitates fertilization in Asian sacred lotus. Annals of Botany, 103(7), 1159–1163. 19282320 10.1093/aob/mcp051 PMC2707905

[ece310319-bib-0035] Maekawa, H. , Otsubo, M. , Sato, M. P. , Takahashi, T. , Mizoguchi, K. , Koyamatsu, D. , Inaba, T. , & Ito‐Inaba, Y. (2022). Establishing an efficient protoplast transient expression system for investigation of floral thermogenesis in aroids. Plant Cell Reports, 41(1), 263–275. 34704119 10.1007/s00299-021-02806-1

[ece310319-bib-0036] Mayo, S. J. , Bogner, J. , Boyce, P. C. , & Boyce, P. J. (1997). The genera of Araceae. Royal Botanic Gardens.

[ece310319-bib-0037] Meeuse, B. J. D. (1975). Thermogenic respiration. Annual Review of Plant Physiology, 26(83), 117–126.

[ece310319-bib-0038] Meeuse, B. J. D. , & Raskin, I. (1988). Sexual reproduction in the arum lily family, with emphasis on thermogenicity. Sexual Plant Reproduction, 1(1), 3–15.

[ece310319-bib-0039] Murray, M. G. , & Thompson, W. F. (1980). Rapid isolation of high molecular weight plant DNA. Nucleic Acids Research, 8(19), 4321–4326. 7433111 10.1093/nar/8.19.4321 PMC324241

[ece310319-bib-0040] Nie, Z. L. , Sun, H. , Li, H. , & Wen, J. (2006). Intercontinental biogeography of subfamily Orontioideae (*Symplocarpus*, *Lysichiton*, and *Orontium*) of Araceae in eastern Asia and North America. Molecular Phylogenetics and Evolution, 40(1), 155–165. 16621613 10.1016/j.ympev.2006.03.012

[ece310319-bib-0041] Otsuka, K. (2002). Distribution of *Symplocarpus* (Araceae) in Japan; especially of *S. nabekuraensis* . Bulletin of Nagano Nature Conservation Research Institute, 5, 1–8.

[ece310319-bib-0042] Otsuka, K. , Hamada, T. , & Ueda, K. (2011). Thermogenesis in Japanese *Symplocarpus* species (Araceae). Journal of Japanese Botany, 86(4), 224–229.

[ece310319-bib-0043] Otsuka, K. , & Kitano, S. (2003). Predation by rodents on fruits and flowers of three species of *Symplocarpus* (Araceae). Bulletin of Nagano Nature Conservation Research Institute, 6, 29–34.

[ece310319-bib-0044] Otsuka, K. , Watanabe, R. , & Inoue, K. (2002). A new species of *Symplocarpus* (Araceae) from Nagano prefecture, Central Japan. Journal of Japanese Botany, 77, 96–100.

[ece310319-bib-0045] Pavlova, N. S. , & Nechaev, V. A. (2005). A new species of the genus *Symplocarpus* (Araceae) from the southern Russian Far East. Botanicheskii Zhurnal, 90(5), 753–758.

[ece310319-bib-0046] Petit, R. J. , Duminil, J. , Fineschi, S. , Hampe, A. , Salvini, D. , & Vendramin, G. G. (2005). Comparative organization of chloroplast, mitochondrial and nuclear diversity in plant populations. Molecular Ecology, 14(3), 689–701. 15723661 10.1111/j.1365-294X.2004.02410.x

[ece310319-bib-0047] Phillips, S. J. , & Dudík, M. (2008). Modeling of species distributions with Maxent: New extensions and a comprehensive evaluation. Ecography, 31(2), 161–175.

[ece310319-bib-0048] Rochette, N. C. , Rivera‐Colón, A. G. , & Catchen, J. M. (2019). Stacks 2: Analytical methods for paired‐end sequencing improve RADseq‐based population genomics. Molecular Ecology, 28, 4737–4754. 31550391 10.1111/mec.15253

[ece310319-bib-0049] Sang, T. , Crawford, D. J. , & Stuessy, T. F. (1997). Chloroplast DNA phylogeny, reticulate evolution, and biogeography of *Paeonia* (Paeoniaceae). American Journal of Botany, 84(8), 1120–1136. 21708667

[ece310319-bib-0050] Schoener, T. W. (1968). Sizes of feeding territories among birds. Ecology, 49(1), 123–141.

[ece310319-bib-0051] Seymour, R. S. (2010). Scaling of heat production by thermogenic flowers: Limits to floral size and maximum rate of respiration. Plant, Cell & Environment, 33(9), 1474–1485. 10.1111/j.1365-3040.2010.02190.x 20545882

[ece310319-bib-0052] Seymour, R. S. , White, C. R. , & Gibernau, M. (2003). Heat reward for insect pollinators. Nature, 426(6964), 243–244. 14628037 10.1038/426243a

[ece310319-bib-0053] Singhal, S. , & Moritz, C. (2012). Testing hypotheses for genealogical discordance in a rainforest lizard. Molecular Ecology, 21(20), 5059–5072. 22989358 10.1111/j.1365-294X.2012.05747.x

[ece310319-bib-0054] Stamatakis, A. (2014). RAxML version 8: A tool for phylogenetic analysis and post‐analysis of large phylogenies. Bioinformatics, 30(9), 1312–1313. 24451623 10.1093/bioinformatics/btu033 PMC3998144

[ece310319-bib-0055] Strijk, J. S. , Binh, H. T. , Van Ngoc, N. , Pereira, J. T. , Ferry Slik, J. W. , Sukri, R. S. , Suyama, Y. , Tagane, S. , Wieringa, J. J. , Yahara, T. , & Hinsinger, D. D. (2020). Museomics for reconstructing historical floristic exchanges: Divergence of stone oaks across Wallacea. PLoS One, 15(5), 1–20. 10.1371/journal.pone.0232936 PMC7244142 32442164

[ece310319-bib-0056] Suyama, Y. , Hirota, S. K. , Matsuo, A. , Tsunamoto, Y. , Mitsuyuki, C. , Shimura, A. , & Okano, K. (2022). Complementary combination of multiplex high‐throughput DNA sequencing for molecular phylogeny. Ecological Research, 37(1), 171–181.

[ece310319-bib-0057] Swets, J. A. (1973). The relative operating characteristic in psychology. Science, 182(4116), 990–1000. 17833780 10.1126/science.182.4116.990

[ece310319-bib-0058] Tanabe, A. S. , & Toju, H. (2013). Two new computational methods for universal DNA barcoding: A benchmark using barcode sequences of bacteria, archaea, animals, fungi, and land plants. PLoS One, 8(10), e76910. 10.1371/journal.pone.0076910 24204702 PMC3799923

[ece310319-bib-0059] Tang, W. (1987). Heat production in cycad cones. Botanical Gazette, 148(2), 165–174. 10.1086/337644

[ece310319-bib-0060] Uemura, S. , Ohkawara, K. , Kudo, G. , Wada, N. , & Higashi, S. (1993). Heat‐production and cross‐pollination of the Asian skunk cabbage *Symplocarpus renifolius* (Araceae). American Journal of Botany, 80, 635–640.

[ece310319-bib-0061] van Proosdij, A. S. J. , Sosef, M. S. M. , Wieringa, J. J. , & Raes, N. (2016). Minimum required number of specimen records to develop accurate species distribution models. Ecography, 39(6), 542–552.

[ece310319-bib-0062] Wada, N. , & Uemura, S. (1994). Seed dispersal and predation by small rodents on the herbaceous understory plant *Symplocarpus renifolius* . The American Midland Naturalist, 132(2), 320–327.

[ece310319-bib-0063] Wagner, A. M. , Krab, K. , Wagner, M. J. , & Moore, A. L. (2008). Regulation of thermogenesis in flowering Araceae: The role of the alternative oxidase. Biochimica et Biophysica Acta – Bioenergetics, 1777(7–8), 993–1000. 10.1016/j.bbabio.2008.04.001 18440298

[ece310319-bib-0064] Warren, D. L. , Glor, R. E. , & Turelli, M. (2008). Environmental niche equivalency versus conservatism: Quantitative approaches to niche evolution. Evolution, 62(11), 2868–2883. 18752605 10.1111/j.1558-5646.2008.00482.x

[ece310319-bib-0065] Warren, D. L. , Matzke, N. J. , Cardillo, M. , Baumgartner, J. B. , Beaumont, L. J. , Turelli, M. , Glor, R. E. , Huron, N. A. , Simões, M. , Iglesias, T. L. , Piquet, J. C. , & Dinnage, R. (2021). ENMTools 1.0: An R package for comparative ecological biogeography. Ecography, 44(4), 504–511.

[ece310319-bib-0066] Xu, L.‐L. , Yu, R.‐M. , Lin, X.‐R. , Zhang, B.‐W. , Li, N. , Lin, K. , Zhang, D.‐Y. , & Bai, W.‐N. (2021). Different rates of pollen and seed gene flow cause branch‐length and geographic cytonuclear discordance within Asian butternuts. The New Phytologist, 232(1), 388–403. 34143496 10.1111/nph.17564 PMC8519134

[ece310319-bib-0067] Yu, G. , Smith, D. K. , Zhu, H. , Guan, Y. , & Lam, T. T. Y. (2017). Ggtree: An R package for visualization and annotation of phylogenetic trees with their covariates and other associated data. Methods in Ecology and Evolution, 8(1), 28–36.

