# Supplementary material for: Potential contribution of floral thermogenesis to cold adaptation, distribution pattern, and population structure of thermogenic and non/slightly thermogenic Symplocarpus species
Source: Ecol Evol. 2023 Jul 15;13(7):e10319. doi: 10.1002/ece3.10319 (PMC10349278; doi:10.1002/ece3.10319)
Supplement: Supplementary file 1 — Figures S1–S5 [file ECE3-13-e10319-s002.docx]

**Supplementary Figure S1**

Comparison of the range of five environmental variables (bio02: Monthly Mean Diurnal Range, bio08: Mean Temperature of Wettest Quarter, bio10: Mean Temperature of Warmest Quarter, bio13: Precipitation of Wettest Month, bio19: Precipitation of Coldest Quarter) used in this study to select climate models with less extrapolation in Mid-Holocene (MH) and Last Glacial Maximum (LGM). Environmental data for MH and LGM were estimated using nine (BCC-CSM1-1: BCMH, CCSM4: CCMH, CNRM-CM5: CNMH, HadGEM2-CC: HGMH, HadGEM2-ES: HEMH, IPSL-CM5A-LR: IPMH, MIROC-ESM: MRMH, MPI-ESM-P: MEMH, and MRI-CGCM3: MGMH) and three models (CCSM4: CCLGM, MIROC-ESM: MRLGM, MPI-ESM-P: MELGM), respectively.

**Supplementary Figure S2**

Predictability indices for five ecological niche modeling methods.

**Supplementary Figure S3**

Mean temperature of the coldest quarter (bio11) at occurrence locations.

**Supplementary Figure S4**

Maxent prediction of potential distributions of *S. nipponicus* (left) and *S. renifolius* (right) around Northeast Asia in current and less extrapolation climate models in the MH (BCMH, CCMH, CNMH, and MEMH) and LGM (MRLGM).

**Supplementary Figure S5**

Cross-validation error of each K (the number of clusters) for population structure analysis using ADMIXTURE.
